# Pregnancy in women diagnosed with HIV on antiretroviral therapy in Ethiopia: a retrospective cohort study

**DOI:** 10.11604/pamj.2020.37.101.23035

**Published:** 2020-09-29

**Authors:** Matilda Elliver, Inger Hallström, Degu Jerene

**Affiliations:** 1 Faculty of Medicine, Lund University, Lund, Sweden,; 2 Koninklijke Centrale Vereeniging (KNCV) Tuberculosis Foundation, The Hague, Netherlands

**Keywords:** Antiretroviral therapy, Ethiopia, HIV, pregnancy, women

## Abstract

**Introduction:**

due to increasing coverage of antiretroviral therapy (ART), more women living with HIV have improved health condition which also increases their chances of getting pregnant. However, the knowledge about pregnancy among women receiving ART in resource-constrained settings, like Ethiopia, is limited. The aim was to assess factors associated with pregnancy among women living with HIV in Ethiopia.

**Methods:**

a retrospective cohort study from 2005 through 2013 including a total of 809 women aged 15-49 years on ART was used. The study was conducted in eight hospitals and health centers in two regions of Ethiopia. The data was collected between March and June of 2014 from patients´ pre-ART and ART registers by trained nurses, assisted by data entry clerks and supervised by senior physicians. Kaplan-Meier survival analysis and Cox regression analysis were used to examine the probability of becoming pregnant. Factors associated with pregnancy were presented with hazard ratios with 95% Confidence Interval (CI).

**Results:**

a total of 809 women were included in this analysis, their median age was 27 years, 90% were urban residents and 40.6% were married. Four hundred eighty three (60.6%) were in WHO stage III at initiation of ART. The median CD4 count was 162.5 cell/μl at initiation of ART. Eighty-one women became pregnant during 3069 person-years of observation. The overall incidence of pregnancy was 26.4 pregnancies per 1000 person-years of observation. Women under the age of 24, those in less advanced disease stage, women with no education and those with college education had higher rates of pregnancy.

**Conclusion:**

the results highlight that younger women, those in less advanced disease stage, either uneducated or highly educated ones have higher probability of becoming pregnant during HIV treatment. This suggests the need for integrating reproductive health services into HIV care services to meet the needs of women on ART.

## Introduction

Human Immunodeficiency Virus (HIV) is still a major global health issue - 36.9 million people are estimated to currently be living with HIV. In Ethiopia, it is estimated that 610,000 people are infected with HIV. The prevalence of HIV among women aged 15-49 in Ethiopia is projected to be 1.4% and that of men to be 0.7% [1]. Antiretroviral therapy (ART) includes three or more antiretroviral (ARV) drugs for treating HIV infection [2]. With ART people diagnosed with HIV can live long and healthy lives with reduced number of coinfections and a 96% reduced risk of transmitting to a sexual partner [3]. A free of charge ART program was launched in Ethiopia in 2005 [4]. Since then, the coverage of people receiving ART has increased significantly, today reaching 65% of women over the age of 15 living with HIV [1]. The fertility desire among women receiving ART varies in different parts of the country. The desire to give birth among HIV infected women receiving ART in Tigray region, Finote Selam in Mirab Gojjam zone in Amhara region, Addis Ababa and South Wollo zone was 45.5%, 28.8%, 44% and 15.7%, respectively [5-8]. The fertility desire among both men and women living with HIV and being treated with ART in Fiche and Addis Ababa was 39.1% and 54.6%, respectively [9,10]. A meta-analysis consisting of twenty studies from Ethiopia, Uganda, South Africa, Nigeria, USA, France, Brazil and Canada showed that age less than 30 years and not having children were strongly associated with fertility desire among HIV positive women [11].

In Jimma town in South West Ethiopia, fertility desire among women receiving ART was associated with being in the age of 18-29 years, being married, having diploma educational level, having only boys or girls children, having 500-1000 Ethiopian Birr as monthly income and having a partner that was not diagnosed with HIV [12]. For women diagnosed with HIV intending to become pregnant it is important to know the risks involved while being HIV positive. Possible risks include mother to child transmission (MTCT) that may arise in utero, during birth or through breastfeeding [13]. The transmission rates vary from 15% - 45% without any intervention. MTCT can be reduced by usage of ART, planned caesarean section and restricted breast feeding to below 5% [3,13]. Knowledge is fundamental to reduce mother-to-child transmission of HIV. However, only 57% of women aged 15-49 in Ethiopia are estimated to have knowledge about the different transmission ways of HIV from mother to child [14]. In 2013, WHO recommends lifelong ART for all pregnant and breastfeeding HIV-infected women, irrespective of WHO clinical stage to prevent MTCT [2]. It is estimated that 59% of pregnant women in Ethiopia receive ARV for prevention of MTCT [15]. WHO additionally advises infants born to mothers diagnosed with HIV to receive ARV prophylaxis the first six weeks of life to prevent MTCT [2].

According to the Ethiopian guidelines, monthly follow up visits are recommended until infection status can be determined for an HIV exposed infant, which is done through deoxyribonucleic acid (DNA) Polymerase Chain Reaction (PCR) and rapid antibody test [4]. A study has shown that there is a higher incidence of spontaneous abortion, stillbirth, perinatal and infant death, low birth weight and chorioamnionitis among pregnant women diagnosed with HIV. In developing countries where the access to specialist care is limited, HIV infection is in fact one of the leading causes of maternal mortality [13]. A systematic review and meta-analysis has also indicated that pregnant women diagnosed with HIV have eight times higher risk of pregnancy-related deaths compared to those not infected with HIV [16]. The number of women living with HIV who are being treated with ART in Ethiopia is increasing. There is limited knowledge about the actual rate of pregnancy among these women. Increased knowledge about pregnancy rate and predictors and/or factors of pregnancy is essential to be able to prevent unintended pregnancies, offer family planning and to understand the appropriate care for women on ART. The aim of this study was to assess rate of and factors associated with pregnancy among women living with HIV and treated with ART in Ethiopia.

## Methods

**Study design:** this was a retrospective cohort study using nine-year data from January 2005 through December 2013. The study was conducted in eight hospitals and health centers in two regions of Ethiopia-Addis Ababa and Southern Nations, Nationalities and People´s Region (SNNPR). Addis Ababa is the capital city and has the lowest prevalence of poverty and the highest median duration of education in the country. HIV prevalence among women aged 15-49 is higher in Addis Ababa compared to SNNPR (4.2% and 0.5%, respectively) [14]. All eight health centers offer ART according to the WHO treatment guidelines and the national guidelines for comprehensive HIV prevention, care and treatment [2,4].

**Participants:** women aged 15-49 years who were enrolled in HIV care between 2005-2012 and followed through end of 2013 at the eight chosen hospitals were taken as the study population. The data was collected between March and June of 2014 from patients´ pre-ART and ART registers by trained nurses, assisted by data entry clerks and supervised by senior paediatricians.

**Statistical analysis**: rate of pregnancy in person-years of follow up was calculated. Kaplan-Meier survival analysis was used to examine the probability of becoming pregnant and factors associated with pregnancy. The variables of the study were age, region, residence, education, marital status and WHO stage, CD4 count, body weight, body mass index (BMI), haemoglobin, total lymphocyte count (TLC) at initiation of ART. Variables with overall comparison p-value <0.25 at Kaplan Meier survival analysis was further analysed using multivariate Cox regression survival analysis and hazard analysis [17]. The reference group was those with the least risk of pregnancy and results were presented with hazard ratios with 95% Confidence Interval (CI). Cox regression was adjusted for age, region, residence, marital status, education and WHO stage, body weight and haemoglobin at initiation of ART. Statistical significance was defined as p-value <0.05. SPSS™ version 25.0 was used for data analysis.

**Ethical considerations:** ethical clearance was obtained in 2013 from the Institutional Review Board of Addis Ababa University, College of Health Sciences (reference number 070/12/SPH), the National Research Ethics Review Committee (reference number 3.10/564/06) and the ethics review committees of Addis Ababa and SNNPR health bureaus.

## Results

Out of 1093 women, 809 (73.9%) were prescribed on ART, 203 (18.6%) were lost to follow up, 37 (3.4%) were still in pre-ART care, 30 (2.7%) were transferred out and 14 (1.3%) were dead. Therefore, a total of 809 women out of 1093 were selected for analysis. Among the 809 women, the median age was 27 years and 737 (91.1%) were from SNNPR. Seven hundred and eleven (90.0%) were urban residents and 321 (40.6%) were married (Table 1). Four hundred eighty three (60.6%) were in WHO stage III at initiation of ART. The median body weight at initiation of ART was 48.0kg. The median BMI was 15.2kg/m^2^ and 365 (87.7%) had a BMI below 18.5kg/m^2^. The median TLC count was 1116.0 cell/μl, the median haemoglobin was 11.3 mg/dl and the median CD4 count was 162.5 cell/μl at initiation of ART (Table 2). The overall incidence of pregnancy was 26.4 pregnancies per 1000 person-years of observation (81 pregnancies in a total of 3069 person-years of observation) among women on ART. Among 59 pregnant women with data on time to pregnancy after enrolment, the median time was 1.2 years. The cumulative probability of surviving from pregnancy was lower among women under the age of 24 compared to women over the age of 24, with a mean survival time of 7.8 and 8.9 years, respectively (95% CI 7.3-8.3 and 95% CI 8.7-9.1, respectively). This shows that the cumulative hazard of pregnancy was higher among women younger than 24 years. The cumulative probability of surviving from pregnancy was lower among women in WHO stage I and II with a mean survival time of 5.3 years (95% CI 4.4-6.3) and 7.4 years (95% CI 6.9-8.0) compared to women in stage III and IV with a mean survival time of 8.7 years (95% CI 8.5-9.0) and 9.3 years (95% CI 8.8-9.9), respectively.

**Table 1:** sociodemographic characteristics of women receiving ART at eight hospitals in Addis Ababa and SNNPR in Ethiopia during the period 2005-2013

Variables	Pregnancy (%)	No pregnancy (%)	Total frequency (%)
**Age**			
15-24 years	48 (15.9)	253 (84.1)	301 (37.5)
25-34 years	32 (10.3)	279 (89.7)	311 (38.7)
35-44 years	1 (0.5)	190 (99.5)	191 (23.8)
**Region**			
Addis Ababa	12 (16.7)	60 (83.3)	72 (9.0)
SNNPR	69 (9.4)	662 (90.6)	731 (91.0)
**Place of residence**			
Urban	78 (11.0)	633 (89.0)	711 (90.0)
Rural	3 (4.8)	76 (96.2)	79 (10.0)
**Marital status**			
Married	47 (14.6)	274 (85.4)	321 (40.6)
Not married	23 (10.3)	200 (89.7)	223 (28.2)
Widowed	1 (1.1)	88 (98.9)	89 (11.2)
Divorced/separated	9 (6.2)	135 (93.8)	144 (18.2)
Not applicable	1 (7.1)	13 (92.9)	14 (1.8)
**Education**			
No formal education	17 (9.7)	159 (90.3)	176 (22.3)
Grade 1-8	41 (13.1)	271 (86.9)	312 (39.5)
Grade 9-12	16 (6.9)	217 (93.1)	233 (29.5)
College and above	5 (11.4)	39 (88.6)	44 (5.5)
No information	1 (4.0)	24 (96.0)	25 (3.2)

**Table 2:** clinical characteristics of women receiving ART at eight hospitals in Addis Ababa and SNNPR in Ethiopia during the period 2005-2013

Variables	Pregnancy (%)		No pregnancy (%)	Total frequency (%)
**WHO stage at initiation of ART**				
WHO stage I	18 (26.5)		50 (73.5)	68 (8.5)
WHO stage II	13 (11.9)		96 (88.1)	109 (13.7)
WHO stage III	43 (8.9)		440 (91.1)	483 (60.6)
WHO stage IV	6 (4.4)		131 (95.6)	137 (17.2)
**Body mass index (BMI)**				
≤18,5 kg/m^2^	27 (7.4)		338 (92.6)	365 (87.7)
>18,5 kg/m^2^	9 (17.6)		42 (82.4)	51 (12.3)
**Body weight at initiation of ART**				
<35 kg	3 (4.5)		63 (95.5)	66 (8.2)
35-50 kg	39 (8.7)		408 (91.3)	447 (55.7)
>50 kg	39 (13.4)		251 (86.6)	290 (36.1)
**Haemoglobin at initiation of ART**				
≤10 mg/dl	14 (6.0)		220 (94.0)	234 (31.8)
>10 mg/dl	63 (12.5)		439 (87.5)	502 (68.2)
**Total lymphocyte count at initiation of ART**				
≤1200 cell/μl	8 (4.4)		172 (95.6)	180 (48.8)
>1200 cell/μl	15 (7.9)		174 (92.1)	189 (51.2)
**CD4 count at initiation of ART**				
≤350 cell/μl	66 (9.7)		612 (90.3)	678 (84.4)
>350 cell/μl	15 (12.0)		110 (88.0)	125 (15.6)
				

**Figure 1:**
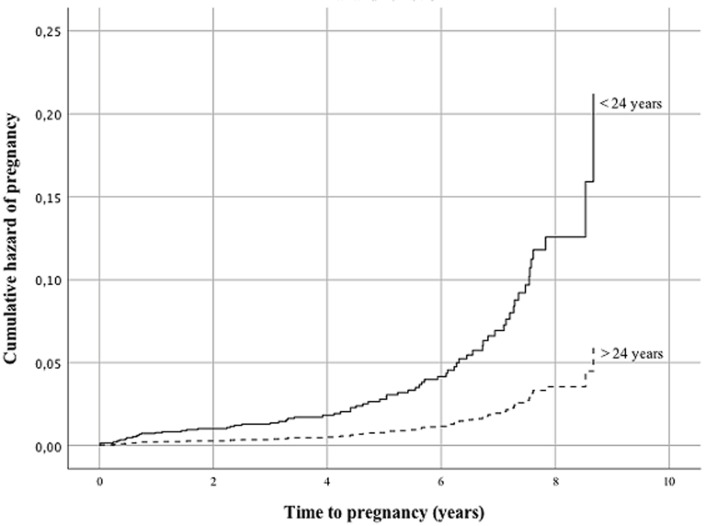
Cox regression hazard function comparing age among women receiving ART between 2005-2013 at eight hospitals in Addis Ababa and SNNPR in Ethiopia

**Figure 2:**
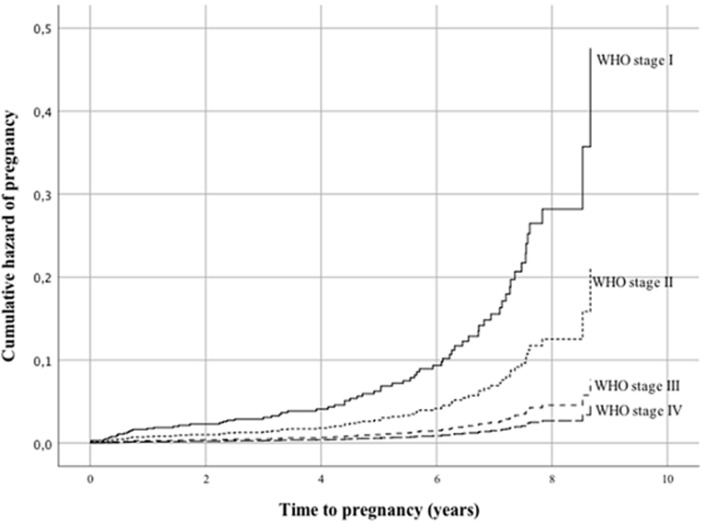
Cox regression hazard function comparing WHO clinical stage among women receiving ART between 2005-2013 at eight hospitals in Addis Ababa and SNNPR in Ethiopia

**Figure 3:**
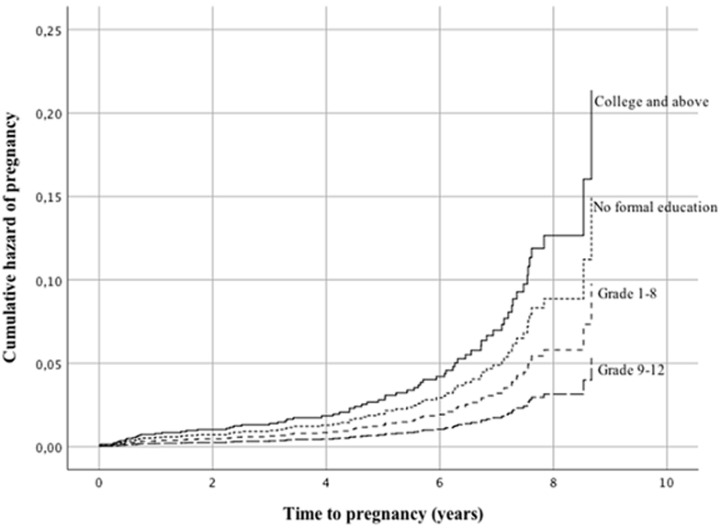
Cox regression hazard function comparing education level among women receiving ART between 2005-2013 at eight hospitals in Addis Ababa and SNNPR in Ethiopia

The cumulative hazard of pregnancy was therefore higher among women in stage I and II. The cumulative probability of surviving from pregnancy was lower among women with BMI ≤18.5 kg/m^2^ with a mean survival time of 9.3 years (95% CI 9.0-9.6) compared to women with BMI >18.5 kg/m^2^ with a mean survival time of 7.7 years (95% CI 7.0-8.3). The cumulative hazard of pregnancy was thus higher among women with a BMI above 18.5 kg/m^2^. The mean survival time from pregnancy was 9.1 years (95% CI 8.7-9.6) and 8.2 years (95% CI 7.9-8.5) for women with haemoglobin ≤10 mg/dl and haemoglobin >10 mg/dl at initiation of ART, respectively. This means the cumulative hazard of pregnancy was higher among women with haemoglobin >10 mg/dl than ≤10 mg/dl. There was no significance in the unadjusted analysis for residence, education, adherence to ART, body weight and CD4 count or TLC at initiation of ART. The odds of getting pregnant among women under the age of 24 years was 3.5 times higher compared to women over the age of 24 years (HR=3.547, 95% CI 2.049-6.139) (Figure 1, Table 3). Women in WHO clinical stage I and II had 10.5 and 4.7 times higher odds of pregnancy compared to women in WHO stage IV (HR=10.495, 95% CI 3.451-31.920 and HR=4.662, 95% CI 1.552-14.009) (Figure 2, Table 3). Women with no formal education and those with college education or above had 2.8 and 4.0 times higher odds of pregnancy compared to women who had finished grade 9-12 (HR=2.811, 95% CI 1.323-5.974 and HR=4.016, 95% CI 1.412-11.425) (Figure 3, Table 3).

**Table 3:** Cox proportional hazard regression of factors associated with pregnancy among women receiving ART at eight hospitals in Addis Ababa and SNNPR in Ethiopia during the period 2005-2013

Variables	Adjusted hazard ratio (95% CI)	p-value
**Age**		
≤24 years	3.547 (2.049-6.139)	0.000
>24 years	1	
**Region**		
Addis Ababa	1.682 (0.771-3.669)	0.191
SNNPR	1	
**Place of residence**		
Urban	1.239 (0.381-4.033)	0.722
Rural	1	
**Education**		
No formal education	2.811 (1.323-5.974)	0.007
Grade 1-8	1.838 (0.987-3.422)	0.055
Grade 9-12	1	
College and above	4.016 (1.412-11.425)	0.009
**WHO stage at initiation of ART**		
WHO stage I	10.495 (3.451-31.920)	0.000
WHO stage II	4.662 (1.552-14.009)	0.006
WHO stage III	1.699 (0.651-4.436)	0.279
WHO stage IV	1	
**Body weight at initiation of ART**		
<35 kg	1	
35-50 kg	1.305 (0.390-4.366)	0.666
>50 kg	2.219 (0.646-7.622)	0.205
**Haemoglobin at initiation of ART**		
≤10 mg/dl	1	
>10 mg/dl	1.545 (0.823-2.900)	0.176

## Discussion

This study found that women on ART in SNNPR and Addis Ababa in Ethiopia had an overall incidence of pregnancy of 26.4 pregnancies per 1000 person-years of observation. Incidence of pregnancy among women on ART has been found to be higher in other studies, such as in North West Ethiopia, Mbarara in Uganda, urban Malawi, Zimbabwe and South Africa with an incidence of 49.2, 94.0, 93, 77 and 52 per 1000 person years, respectively [18-22]. This difference might be due to different length of the study period, the size of the study and also that fertility rates may differ in different countries and regions. In the unadjusted analysis, age under 24 years, being in WHO clinical stage I or II, BMI >18.5 kg/m^2^ and haemoglobin >10 mg/dl was shown to be associated with a higher probability of pregnancy. Studies from Malawi and Uganda also showed that women with a BMI >18.5 kg/m^2^ were more likely to become pregnant than those with a BMI ≤18.5 kg/m^2^ [20,23]. Because of missing data, BMI was not further analysed in the present study. Both WHO clinical stage, BMI and haemoglobin level reveal something about the progression of the HIV infection and the health of the individual [24,25], which would most likely affect pregnancy rate.

In our study, the chance of getting pregnant among women under the age of 24 years was 3.5 times higher than for women over the age of 24 years. A study from Mbarara, Uganda also found that younger age was associated with an increased probability of pregnancy, as well as studies from South Africa, Malawi and western Uganda [19,20,22,26]. The explanation is probably that it is more common for women overall to have children at a younger age in Ethiopia and the fact that younger women are generally more fertile. Women in WHO stage I and II at initiation of ART had 10.5 and 4.7 times higher odds of becoming pregnant compared to women in WHO stage IV. A study in Malawi reported the same findings [20]. This might be due to the fact that women in stage I and II at initiation of ART have a better health and have not had many coinfections, which probably influences both the fertility and the fertility desire. In a study in western Uganda WHO stage was not a significant predictor of pregnancy [26]. This difference in results might be due to different length of the study period and the size of the study. The probability of pregnancy was 4.0 and 2.8 times higher among women with college education or above and women with no formal education, respectively, compared to women who had finished grade 9-12. The explanation to this might be that women with college education or above probably have more knowledge about their HIV-infection and that mother to child transmission can be reduced significantly when following the WHO guidelines. The reason for women with no formal education to have a higher risk of pregnancy might be due to pressure from the society or husband to have children, since children are seen as an asset in Ethiopia. Another reason might be that they do not have access to family planning or as much knowledge about the different transmission ways of HIV. This finding was not in line with findings from a study in western Uganda where primary education was associated with a 1.65 times higher risk of pregnancy compared to those with tertiary education. Those with no education and those with college education were not of higher risk of pregnancy [26]. This discrepancy might be explained by different length of the study period and the size of the study, but should be further studied.

In the multivariable analysis, region, place of residence, marital status, body weight, haemoglobin and TLC at initiation of ART were not found to be significantly associated with pregnancy. A study in Uganda indicated that married and single women were more likely to become pregnant compared to widowed or separated [26], which was not found in the present study. A study in northwest Ethiopia found that rural, married, widowed and divorced, unemployed women and women with less than two children were more likely to become pregnant compared to their counterparts [18]. This was not found in the present study, which may be due to different length of the study period, the size of the study and that some of these variables were not included in the material in this study. The fact that 91.0% were from SNNPR and that 90.0% were urban residents most likely affects the results. HIV is predominately an urban disease in Ethiopia and the result may be difficult to generalise to other populations. A limitation of the present study is the low number of participants, which should be extended in future studies. Some of the variables could not be further analysed in multivariable Cox regression because of too many missing data due to the retrospective nature of the study. In addition to this, interesting variables such as use of family planning, fertility desire, intended and unintended pregnancies, number of children prior to the pregnancy were not included. Long-term follow-up of HIV exposed infants for potential birth defects is another area for future studies, which would require establishing birth registries at several sites. A strength of the study was that participants were followed through a long period of time - a total of nine years.

## Conclusion

The incidence of pregnancy among women diagnosed with HIV on ART is considerable. The results point to the importance of focusing on certain groups of women diagnosed with HIV on ART who have an increased probability of pregnancy. Integration of reproductive health services to HIV care services to prevent unintended pregnancies, prevent MTCT and meet the needs of women on ART is of greatest importance.

### What is known about this topic

- ART use is associated with significantly higher rates of pregnancy among people living with HIV;
- Lower educational status is associated with higher risk of pregnancy during ART.

### What this study adds

- Extremes of educational status (either in the highest or lowest end) are associated with increased risk of pregnancy during ART;
- Women in less advanced disease stage are at higher risk of becoming pregnant during ART follow-up.
